# Survival benefit of helicopter emergency medical services compared to ground emergency medical services in traumatized patients

**DOI:** 10.1186/cc12796

**Published:** 2013-06-21

**Authors:** Hagen Andruszkow, Rolf Lefering, Michael Frink, Philipp Mommsen, Christian Zeckey, Katharina Rahe, Christian Krettek, Frank Hildebrand

**Affiliations:** 1 Department of Trauma and Reconstructive Surgery, University Hospital Aachen, Pauwelsstraße 30, 52074 Aachen, Germany; 2 Institute for Research in Operative Medicine (IFOM), University of Witten/Herdecke, Ostmerheimer Str. 200, 51109 Cologne, Germany; 3 Department for Trauma, Hand and Reconstructive Surgery, University Medical Center Marburg, Baldingerstr., 35043 Marburg, Germany; 4 Trauma Department, Hannover Medical School, Carl Neuberg-Str. 1, 30625 Hannover, Germany

## Abstract

**Introduction:**

Physician-staffed helicopter emergency medical services (HEMS) are a well-established component of prehospital trauma care in Germany. Reduced rescue times and increased catchment area represent presumable specific advantages of HEMS. In contrast, the availability of HEMS is connected to a high financial burden and depends on the weather, day time and controlled visual flight rules. To date, clear evidence regarding the beneficial effects of HEMS in terms of improved clinical outcome has remained elusive.

**Methods:**

Traumatized patients (Injury Severity Score; ISS ≥9) primarily treated by HEMS or ground emergency medical services (GEMS) between 2007 and 2009 were analyzed using the TraumaRegister DGU^® ^of the German Society for Trauma Surgery. Only patients treated in German level I and II trauma centers with complete data referring to the transportation mode were included. Complications during hospital treatment included sepsis and organ failure according to the criteria of the American College of Chest Physicians/Society of Critical Care Medicine (ACCP/SCCM) consensus conference committee and the Sequential Organ Failure Assessment (SOFA) score.

**Results:**

A total of 13,220 patients with traumatic injuries were included in the present study. Of these, 62.3% (*n *= 8,231) were transported by GEMS and 37.7% (*n *= 4,989) by HEMS. Patients treated by HEMS were more seriously injured compared to GEMS (ISS 26.0 vs. 23.7, *P *< 0.001) with more severe chest and abdominal injuries. The extent of medical treatment on-scene, which involved intubation, chest and treatment with vasopressors, was more extensive in HEMS (*P *< 0.001) resulting in prolonged on-scene time (39.5 vs. 28.9 minutes, *P *< 0.001). During their clinical course, HEMS patients more frequently developed multiple organ dysfunction syndrome (MODS) (HEMS: 33.4% vs. GEMS: 25.0%; *P *< 0.001) and sepsis (HEMS: 8.9% vs. GEMS: 6.6%, *P *< 0.001) resulting in an increased length of ICU treatment and in-hospital time (*P *< 0.001). Multivariate logistic regression analysis found that after adjustment by 11 other variables the odds ratio for mortality in HEMS was 0.75 (95% CI: 0.636 to 862).

Afterwards, a subgroup analysis was performed on patients transported to level I trauma centers during daytime with the intent of investigating a possible correlation between the level of the treating trauma center and posttraumatic outcome. According to this analysis, the Standardized Mortality Ratio, SMR, was significantly decreased following the Trauma Score and the Injury Severity Score (TRISS) method (HEMS: 0.647 vs. GEMS: 0.815; *P *= 0.002) as well as the Revised Injury Severity Classification (RISC) score (HEMS: 0.772 vs. GEMS: 0.864; *P *= 0.045) in the HEMS group.

**Conclusions:**

Although HEMS patients were more seriously injured and had a significantly higher incidence of MODS and sepsis, these patients demonstrated a survival benefit compared to GEMS.

## Introduction

In the prehospital setting, helicopters have been used to transport trauma patients for the past 40 years despite inconsistent evidence of the benefits of helicopter emergency medical systems (HEMS) in civilian trauma systems [1-5]. Since the introduction of helicopters into the civilian trauma system in the 1970s, an ongoing controversy has been provoked as to whether potential benefits outweigh the associated costs [2]. In Germany, a dense network of emergency medical services, including rescue helicopter bases, covers Germany nationwide [6]. Contrary to other countries, HEMS in Germany is exclusively physician-staffed [7]. Therefore, this rescue system is connected to a high financial burden discussed for its presumable benefits [6]. In general, the benefits of HEMS compared to ground emergency medical systems (GEMS) could be: first, transporting a medical team experienced in managing trauma patients. HEMS is commonly accepted to allow a small number of highly skilled and experienced healthcare professionals to perform advanced lifesaving procedures for patients with traumatic injuries [1,8]. Second, facilitating rapid transport from the scene to the hospital based on increased transport velocity has been discussed as an additional benefit of HEMS [1]. Especially so, as helicopters can fly directly to the scene, cover long distances and transport patients from areas inaccessible by ground vehicles, thereby providing severely injured trauma patients with an opportunity to gain access to high level trauma care when this care would otherwise not be in close proximity [9]. Improved triaging of traumatized patients has been mentioned as a third benefit. As HEMS has the ability to travel greater distances, HEMS might be suggested to transport patients directly to a specialist trauma center where definitive treatment can be guaranteed and secondary transfers are avoided [1,2].

Despite the aforementioned aspects, the current literature on the effect of HEMS transport on posttraumatic mortality shows varying results, with several studies finding no significant benefits [5,8]. Contrary findings are suggesting that helicopter transport can decrease mortality [4,10-14]. However, all currently available studies have been conducted in different countries with different emergency services [1]. Furthermore, divergent study methologies and the number of included patients aggravate confident recommendations. The objective of the present study was to evaluate potential benefits of HEMS versus GEMS by analyzing a large number of traumatized patients according to an established trauma registry. We defined in-hospital mortality as a primary outcome of interest to question HEMS' potential benefit. As an additional endeavor, we intended to address the pervading difficulties in drawing inferences from on-scene interventions and transportation mode about mortality by analyzing on-scene management and the accuracy of suspected diagnoses between HEMS and GEMS. Furthermore, incidences of in-hospital complications were evaluated in order to describe the clinical course.

## Materials and methods

### The TraumaRegister Deutsche Gesellschaft für Unfallchirurgie (DGU)^®^

The TraumaRegister DGU^® ^of the German Society for Trauma Surgery (TR-DGU) was established in 1993 and prospectively collects data from more than 300 European trauma centers. Approximately 100 data elements are collected per patient structured in four sections corresponding to the consecutive phases of acute trauma care: A - preclinical phase: mechanism of injury, initial physiology, first therapy, neurological sign and rescue time; B - emergency room: physiology, laboratory findings, diagnostics and interventions; C - intensive care unit: status on admission, organ failure, duration of ventilation; D - final outcome: duration of hospital stay, survival, complete list of injuries and operative procedures. Data are submitted to a central web-based database that is hosted by AUC (Akademie der Unfallchirurgie GmbH) of the DGU. Data are collected on an anonymous basis. Since the TR-DGU is a compulsory tool for quality assessment in German trauma networks, no informed consent was required for data collection. In general, data are available for research purposes after consent by the TraumaRegister DGU^® ^of the German Society for Trauma Surgery (TR-DGU). The investigation was conducted in conformity with ethical principles of research.

### Inclusion criteria

The presented study considered the following patients from the TR-DGU:

- Those treated in a German trauma center level I or II

- Transportation either by helicopter (HEMS) or ground emergency medical services (GEMS), both attended by a physician

- Direct transport from the scene of injury

- Date of admission from January 2007 to December 2009

- Injury Severity Score (ISS) ≥9 points

### Clinical course and assessment of mortality risk

The severity of individual injuries as well as the overall injury severity (Injury Severity Score; ISS) was determined with the Abbreviated Injury Scale (AIS), Revision 2005 [15]. Clinical course included the duration of mechanical ventilation as well as the length of intensive care unit and overall hospital stay. Complications during hospital treatment included sepsis and organ failure. The diagnosis of sepsis was made according to the criteria of the ACCP/SCCM consensus conference committee [16,17]. Organ function status was evaluated according to the Sequential Organ Failure Assessment (SOFA) score [18]. With three or more points, an organ function was considered as failure while multiple organ dysfunction syndrome (MODS) was defined as simultaneous failure of at least two organs.

Since the study groups (HEMS vs. GEMS) were not directly comparable, we used prognostic scores to adjust the observed mortality rates. The prognosis of trauma patients was estimated using the Trauma and Injury Severity Score (TRISS) and the Revised Injury Severity Classification (RISC) [19,20]. TRISS is a logistic regression model that compares outcomes to a large cohort of patients in the Major Trauma Outcomes Study (MTOS), including physiological parameters, trauma mechanism and age [19]. The RISC score is based upon the TraumaRegister DGU^® ^of the German Society for Trauma Surgery (TR-DGU), which analyzes the injury severity and distribution, physiological parameters, and reanimation in order to generate the risk of mortality [20]. While the TRISS was based on pre-hospital data only (blood pressure, consciousness, respiratory rate), the RISC score also considered initial laboratory findings in the emergency department. The prognosis calculated with the TRISS and the RISC method was compared to the actually observed in-hospital mortality rate by calculating the observed vs. expected ratio (Standardized Mortality Ratio, SMR). SMR values were given with 95% confidence intervals (CI) based on the respective CIs of the observed mortality rates. Differences of SMRs were evaluated with the *t*-test. Since the database on which both scores are based are more or less outdated, the SMR itself might be of limited use but interpretation should focus on the relative effects of HEMS vs. GEMS [21].

Multivariate logistic regression analysis with hospital mortality as the dependent endpoint was performed in order to adjust for confounding variables. Besides the mode of transportation, the following variables were considered as confounders in the model: ISS, age, child (age <16 years), unconsciousness (Glasgow Coma Scale; GCS ≤8), shock (prehospital systolic blood pressure ≤90 mmHg), intubation, gender, type of injury (blunt/penetrating), mechanism of injury, level of care of the target hospital, and daytime. Result was reported as odds ratio (OR) with 95% confidence interval.

### Preclinical diagnosis, treatment and mission times

The accuracy of suspected diagnoses during resuscitation was evaluated based on emergency physicians' preclinical documentation of suspected injuries compared to the diagnoses documented clinically in the patients' charts (AIS severity ≥1). The accuracy was described as sensitivity, specificity and positive predictive value in seven different body regions. The sensitivity is defined as the percentage of patients with a respective injury identified by the emergency physician. Specificity is the correctness in patients without that injury. The positive predictive values describe the correctness of the physicians' suspection.

Considerable procedures of on-scene treatment were documented in order to determine potential differences of management skills between HEMS and GEMS.

In addition, the preclinical time (on-scene, transportation and overall rescue time) was analyzed. On-scene time was defined from arrival to abandonment of the scene while overall time was measured from incoming alarm-call to arrival at the emergency room. The duration from on-scene departure to hospital admission was noted as transportation time.

### Subgroup analysis emphasizing on level I trauma centers

A subgroup analysis was performed on patients primarily transported to level I trauma centers during the daytime. This analysis intended to investigate a possible correlation between the level of the treating trauma center and posttraumatic outcome [8]. Furthermore, the presented results referred to rescue efforts in daytime because helicopters are commonly not available after sunset. Daytime was defined as transport that reached the hospital between 6 a.m. and 8 p.m. The subgroup analysis focused on injury severity, complications and outcome.

### Statistics

Incidences were presented with counts and percentages while continuous values were presented as mean and standard deviation (SD) and median with interquartile ranges (IQR 25 to 75) if applicable. Differences between the groups were evaluated with the Wilcoxon rank sum test for continuous data, while Pearson's chi-squared-test was used for categorical variables. A two sided *P*-value < 0.05 was considered to be significant. However, interpretation of data should focus on clinically relevant differences rather than on significant *P*-values.

The data were analyzed using the Statistical Package for the Social Sciences (SPSS; version 20; IBM Inc., Somers, NY, USA).

## Results

### Demographic data

**Figure 1:**
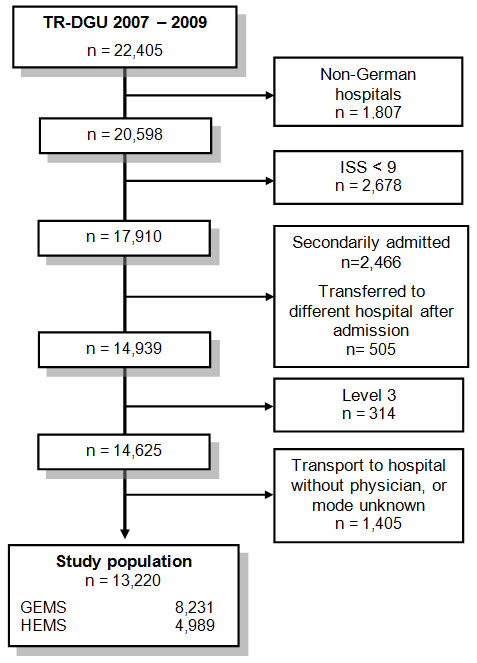
**Study flow chart illustrating and detailing the stratification and selection of patients**.

A total of 13,220 patients were included in the present study (Figure 1). A total of 4,989 (37.7%) patients were transported by HEMS and 8,231 (62.3%) by GEMS. The majority of cases (*n *= 10,742; 81.3%) were brought into a level 1 hospital. The mean age for all patients was 44.4 ± 21.0 years, and 72.8% were male. Patients transported by HEMS were younger (HEMS: 43.1 ± 20.3 years; GEMS: 45.2 ± 21.4; *P *< 0.001) and were more often of male gender (HEMS: 74.8%; GEMS: 71.5%; *P *< 0.001). Nevertheless, comparable trauma cases of children (age <16 years) were transported by HEMS and GEMS (4.8% vs. 4.0%; *P *> 0.05)

### Cause of injury, injury distribution and injury severity

Analyzing the cause of injury, HEMS-transported patients suffered from more high-energy accidents, mainly traffic accidents by car and motorcycle. GEMS-transported patients sustained more low-energy trauma and urban pedestrian accidents (Table 1). Patients treated by HEMS had a significantly higher overall injury severity, emphasizing the chest, extremities and abdominal injuries (Table 2).

**Table 1:** Cause of injury by transportation mode

	HEMS	GEMS	*P*-value
**Car accident**	33.1%	25.3%	<0.001
**Motorcycle accident**	20.3%	12.1%	<0.001
**Bicycle accident**	7.4%	7.7%	0.520
**Pedestrian traffic accident**	4.2%	10.9%	<0.001
**Height fall >3 m**	16.9%	18.9%	0.004
**Height fall <3 m**	7.6%	13.2%	<0.001

**Others**	**10.5%**	**11.9%**	**0.014**

**Table 2:** Injury distribution and injury severity

Number of patients with AIS ≥3	HEMS	GEMS	*P*-value
**Head**	48.2%	47.5%	0.423
**Chest**	54.4%	47.9%	<0.001
**Abdomen**	17.2%	15.3%	0.004
**Extremities**	39.1%	33.3%	<0.001

**ISS**			
**(mean ± SD)**	26.0 ± 13.8	23.7 ± 13.1	
**(median (IQR 25 to 75))**	24 (16 to 34)	21 (14 to 29)	<0.001

### On-scene treatment, rescue times and hospital admission

More preclinical interventions were found in HEMS transported patients (Table 3). On-scene time was greater in HEMS (HEMS: 39.5 ± 21.3 minutes vs. GEMS: 28.9 ± 15.9 minutes; *P *< 0.001). Furthermore, transportation time (HEMS: 20.0 ± 12.3 minutes vs. GEMS: 18.0 ± 13.3 minutes; *P *< 0.001) as well as the overall rescue time (HEMS: 79.9 ± 35.5 minutes vs. GEMS: 62.8 ± 35.1 minutes; *P *< 0.001) were increased.

**Table 3:** On-scene treatment

	HEMS	GEMS	*P*-value
**Intubation**	65.7%	40.6%	<0.001
**Treatment with vasopressors**	10.4%	7.1%	<0.001
**Chest tube**	9.3%	2.7%	<0.001
**Reanimation**	3.2%	3.9%	0.031
**Sedation**	77.2%	64.4%	<0.001

**Volume application**	90.5%	90.9%	0.346

Significant differences for the sensitivity of suspected diagnoses made on-scene referring to the transportation mode were only found for the abdominal region (Table 4). The specificity of suspected diagnoses was significantly better for some body regions in GEMS patients (Table 4).

**Table 4:** Accuracy of suspected diagnoses during resuscitation based on data of 4,049 HEMS and 6,551 GEMS patients with emergency physicians' preclinical documentation of suspected injuries, respectively

	Sensitivity			Specificity			Positive predictive value		
	
	HEMS	GEMS	*P*-value	HEMS	GEMS	*P*-value	HEMS	GEMS	*P*-value
Head	88.9%	88.9%	0.99	60.4%	65.8%	<0.001	78.1%	82.2%	<0.001
Chest	68.4%	67.0%	0.22	71.5%	74.8%	0.022	81.4%	79.4%	0.075
Abdomen	51.5%	55.8%	0.032	74.9%	79.1%	<0.001	40.9%	44.6%	0.044
Upper extremity	63.2%	63.7%	0.74	80.1%	80.6%	0.61	70.7%	67.4	0.030
Lower Extremity	79.7%	79.3%	0.75	84.2%	85.3%	0.25	78.5%	77.6%	0.48
Spine	55.9%	55.8%	0.94	75.4%	80.4%	<0.001	56.7%	56.3%	0.83
**Pelvis**	54.8%	56.8%	0.37	83.8%	86.3%	0.002	49.9%	51.7%	0.36

HEMS patients were more often transported to level I trauma centers compared to GEMS (HEMS: 90.1% vs. GEMS: 75.9%). Accordingly, GEMS transported their patients more frequently to level II (HEMS: 9.9% vs. GEMS: 24.1%).

### Posttraumatic complications, clinical treatment and outcome

Patients treated by HEMS teams had a significantly higher incidence of MODS (HEMS: 33.4% vs. GEMS: 25.0%; *P *< 0.001) and sepsis (HEMS: 8.9% vs. GEMS: 6.6%, *P *< 0.001).

Duration of ventilation (HEMS: 6.8 ± 11.5 days vs. GEMS: 4.9 ± 9.3 days; *P *< 0.001), ICU treatment (HEMS: 10.9 ± 13.7 days vs. GEMS: 8.8 ± 11.9 days; *P *< 0.001) and overall length of stay in hospital (HEMS: 26.2 ± 28.4 vs. GEMS: 21.6 ± 21.9 days; *P *< 0.001) were prolonged following transportation by HEMS. According to the TRISS method (*n *= 7,416), the expected mortality rate was higher than that observed in HEMS patients. Therefore, a significantly decreased SMR was found for these patients (Table 5).

**Table 5:** Survival benefit of HEMS measured by TRISS and RISC

	HEMS	GEMS	*P*-value
**TRISS**			
**Number of cases**	2,949	4,467	
**Expected mortality**	20.4%	17.5%	-
**Observed mortality**	13.8%	14.4%	
**Standardized Mortality Ratio (95% CI)**	0.678 (0.617 to 0.739)	0.825 (0.766 to 0.884)	0.0011
**RISC**			
**Number of cases**	4,575	7,469	
**Expected mortality**	18.3%	17.2%	-
**Observed mortality**	14.6%	14.9%	
**Standardized Mortality Ratio (95% CI)**	0.798 (0.742 to 0.854)	0.869 (0.822 to 0.916)	0.062

Referring to the RISC score (*n *= 12,044), the expected mortality rate tended to be higher compared to the observed mortality in HEMS (Table 5).

### Subgroup analysis: Level I trauma centers

A total of 7,807 patients were transported during daytime to a level I trauma center. A total of 3,855 (49.4%) patients were transported by HEMS and 3,952 (50.6%) by GEMS.

Mean ISS was 26.0 ± 13.7 in HEMS and 24.1 ± 13.3 in GEMS (*P *< 0.001). Time on-scene (HEMS: 39.0 ± 20.2 minutes vs. GEMS: 28.4 ± 15.9 minutes; *P *< 0.001) as well as the overall interval from alarm to hospital admission (HEMS: 78.5 ± 33.1 minutes vs. GEMS: 61.1 ± 32.4 minutes; *P *< 0.001) were enhanced in HEMS. Patients treated by HEMS developed MODS more frequently (HEMS: 33.9% vs. GEMS: 26.4%; *P *< 0.001) while no significant difference was found for the incidence of sepsis (HEMS: 8.5% vs. GEMS: 7.3%; *P *= 0.058).

According to the TRISS method (*n *= 4,450) and the RISC score (*n *= 7,297) a higher mortality rate was expected in HEMS patients (Table 6). Based on the observed mortality rates, significantly decreased SMR was demonstrated in HEMS (SMR TRISS: *P *= 0.002; SMR RISC: *P *= 0.045) (Table 6).

**Table 6:** Survival benefit of HEMS measured by TRISS and RISC in the subgroup of level I trauma centers at daytime

	HEMS	GEMS	*P*-value
**TRISS**			
**Number of cases**	2,294	2,156	
**Expected mortality**	20.7%	18.1%	-
**Observed mortality**	13.4%	14.7%	
**Standardized Mortality Ratio (95% CI)**	0.647 (0.579 to 0.714)	0.815 (0.732 to 0.897)	0.002
**RISC**			
**Number of cases**	3,577	3,720	
**Expected mortality**	18.4%	17.9%	-
**Observed mortality**	14.2%	15.5%	
**Standardized Mortality Ratio (95%-CI)**	0.772 (0.710 to 0.834)	0.864 (0.799 to 0.928)	0.045

Only cases with sufficient data for calculation of score values were considered.

### Outcome benefit of HEMS

Multivariate logistic regression analysis performed in 11,198 cases found that after adjustment by 11 other variables, the OR for mortality in HEMS was 0.75 (95% CI: 0.636 to 862).

## Discussion

Prehospital trauma care is still a matter of ongoing debate with inconsistent evidence comparing the impact of helicopter and ground emergency transport on outcome of traumatized patients. We performed a study comparing the effects of HEMS and GEMS on outcome after trauma. We were able to demonstrate that transportation by HEMS resulted in a significant survival benefit compared to GEMS patients despite increased injury severity and incidence of posttraumatic complications (MODS, sepsis). Sensitivity and specificity of preclinical diagnoses were not superior in HEMS compared to GEMS. The extent of preclinical management was more extensive in HEMS resulting in prolonged on-scene times. Finally, HEMS patients were more often admitted to level I trauma centers.

The most important aspect between HEMS and GEMS to focus on in trauma patients has been the in-hospital mortality. In this respect, the TRISS method has been established as a prognostic tool in several studies. As one of the first studies Baxt *et al*. elucidated a 21% to 50% reduction in TRISS predicted mortality in the 1980s [10,12]. In accordance, Bartolacci *et al*. demonstrated a 50% reduction of mortality by HEMS transportation in patients with an ISS >14 according to the TRISS prediction [22]. In a comparable way to the presented results, Frink *et al*. were able to elucidate a survival benefit of helicopter transported patients [23]. The authors measured the difference between the TRISS-expected and observed mortality finding a considerable observed mortality reduction in HEMS patients while the expected mortality was comparable between the different transportation platforms. Contrary perceptions towards helicopter transportation in traumatized patients was evaluated by Biewener *et al*. [8]. Using the TRISS method with prehospital parameters similar to the presented study, the authors demonstrated no differences between the expected and observed mortality rates between GEMS and HEMS. The authors were not able to reveal that helicopter transport had an impact on mortality outcome but the level of hospital treatment reduced mortality rates markedly. In accordance with Biewener *et al*., Nicholl *et al*. measured no evidence that helicopter rescue improved the chance of survival based upon the TRISS method [24]. However, both studies differ considerably from the presented analysis because less than 1,000 patients were included and only one helicopter station was analyzed, restricting general perceptions. However, according to the presented results, we supported the majority of studies demonstrating a survival benefit [10-12,22,23,25,26]. Although the TRISS method remains the most commonly used tool for benchmarking trauma fatality outcome, its database might be interpreted as outdated and, therefore, should be interpreted carefully [27]. Besides the TRISS based upon prehospital evaluated parameters, we decided to also analyze the RISC score. This score was based upon a more current database including physiological parameters measured on admission [20]. Therefore, differences with respect to the expected mortality rates were found in this study with the RISC score being more accurate compared to the TRISS [20]. However, due to the fact that both scoring systems might potentially be outdated, we were able to support the suspected outcome benefits to HEMS patients by performing a multivariate regression, including multiple potential confounding factors. According to our results, helicopter transport was associated with a significantly reduced mortality risk of 25%. Comparable rates of improved survival have currently been found by Galvagno *et al*. [4]. The authors analyzed the largest study population of approximately 230,000 patients. After adjustment for several confounding factors, helicopter transport was associated with an improved survival of 16% in level I trauma centers and 15% in level II trauma centers.

However, the outcome benefit dependent on the transportation mode seems to be influenced by several aspects, such as on-scene treatment, on-scene time and triage aspects that have to be discussed subsequently [8,13,28,29]. In general, HEMS transport is commonly expected to expedite transport of patients from the scene of an accident to hospital [1,2]. As helicopters are capable of higher speeds over long distances, avoiding difficult terrain, HEMS is expected to support the tenet of trauma management so that the benefit increases considerably when care is delivered within the "golden hour" [28,30,31]. Consequently, a mean overall rescue time of 80 minutes in HEMS patients in this and other research findings [32,33] has to be discussed critically. Despite the results by Newgard *et al*. [33], elucidating no influence of preclinical duration exceeding 60 minutes, and Ringburg *et al*. [29], finding that any influence of prolonged prehospital times was not proven, prolonged on-scene times should be interpreted carefully. It might be argued that longer distances due to transportation to more remote level I trauma centers prolonged the preclinical time in HEMS patients. As transportation times of HEMS were increased in the present study, it could be assumed that travelling distances were enlarged due to a higher rate of primary admission to level I trauma centers in the HEMS group. However, no information about the travelled distances was available in this and other studies [9,29,32]. Therefore, this explanation remains entirely speculative. The aforementioned authors [29,33] argued that the prolonged pre-hospital time might be caused by additional on-scene treatment. Therefore, the potential survival benefit in HEMS has been suggested to depend on rescue teams possessing superior experience in managing trauma patients resulting in extended preclinical procedures [1,8,11]. In order to verify this issue, we measured the extent of on-scene management, on-scene time and the accuracy of suspected diagnoses in physician-staffed HEMS and GEMS [1]. As physician-staffed HEMS and GEMS were compared directly in the present study, we believe that the confounding factor of interpreting preclinical management between different rescue teams (physicians, specialized nurses and paramedics) was addressed adequately. We were able to demonstrate an extended on-scene treatment in HEMS patients as a potential survival benefit. In this context the impact of prehospital intubation in unconscious patients, for example, with severe traumatic brain injury, hemorrhagic shock and respiratory insufficiency is still controversially discussed [32,34]. In the USA, the success of paramedic performed rapid sequence intubation has been shown to depend on the intubation technique and ventilation mode (hyperventilation leading to an increased mortality) and the experience of the performance [34]. On the other side, Miraflor *et al*. currently showed an increased mortality in moderately, initially stable patients with an ISS ≤20 with delayed endotracheal intubation [35]. However, comparability to the presented study might be restricted due to the different health care systems with paramedics performing on-scene management in the USA and physicians performing procedures in Germany. Nevertheless, early intubation as well as the placement of chest tubes could have contributed to a favorable outcome in this study as HEMS patients had an increased incidence of severe chest injuries associated with respiratory insufficiency and a concomitant ISS >25 [36].

Beside the general influence of injury distribution and severity on prehospital treatment [37], the helicopter platform itself was suggested to increase on-scene management: Nakstad *et al*. have demonstrated an increase of intubation rate from 8.2% to 90.2% between ground and helicopter emergency service based on the same indications for endotracheal intubation [32]. Furthermore, Biewener *et al*. revealed an increased incidence of invasive airway management (91% vs. 75%) as well as chest tube insertion (25% vs. 6%) in HEMS [8]. Comparable to the recent study, the authors measured only physician-performed interventions. However, comparability between these studies might be limited as Nakstad *et al*. only analyzed the initial GCS while Biewener *et al*. described their patients by an ISS-based polytrauma degree.

One might conclude that HEMS' physicians diagnose injuries more accurately compared to their grounded colleagues resulting in increased management. Following this hypothesis, we investigated the accuracy of on-scene diagnoses by comparing the sensitivity and specificity in correlation to the clinical diagnoses. In general, predicting the prehospital injury pattern for many injury patterns is known to be difficult and less reliable [38]. In accordance, we did not find a significant difference for the diagnostic accuracy between HEMS and GEMS with the exception of the abdominal region. This might be explained by the fact that especially the abdominal examination on-scene does not reliably detect all patients with intra-abdominal injuries, whereas a relevant number of patients with abdominal pain have no traumatic injuries [39]. However, the accuracy of preclinical diagnoses seemed not to influence the measured survival benefit of HEMS patients as it was demonstrated equally between HEMS and GEMS rescues.

Beside the extent of preclinical procedures, the quality of prehospital management might be assessed by a correct triage of trauma patients with an associated transport to an adequate trauma center [1,2]. Furthermore, studies have already shown a significantly improved survival of trauma patients admitted directly to level I trauma centers [40,41]. Biewener *et al*., therefore, concluded that the level of primary hospital treatment, but not the transportation mode, influenced patients' survival [8]. In order to clarify this issue, we performed a subgroup analysis including patients treated at level I centers and admitted in the daytime. In contrast to Biewener *et al*., an improved survival was observed in HEMS compared to GEMS patients. Consequently, HEMS seemed to influence survival independently of level I treatment.

The aforementioned studies revealing survival benefit of HEMS patients could be criticized due to missing clinical data [3,11-13,22,25,29]. Difficulties remain in drawing conclusions from on-scene risk prognosis to outcome. Especially as complications during the clinical course (for example, MODS and sepsis) considerably determine patients' outcome [42,43]. To address this issue adequately, clinical complications as well as duration of ICU and hospital treatment were evaluated. In this study, HEMS patients required prolonged intensive care treatment and a longer overall length of stay than GEMS patients. This might be explained by the increased ISS of HEMS patients and the associated higher incidences of sepsis and MODS [42,43]. Analyzing the National Trauma Databank (NTDB), Brown *et al*. also found an increased duration of ICU treatment and mechanical ventilation in HEMS patients [28]. The authors also justified this aspect by the concomitant increased injury severity (ISS 15.9 vs. 10.2) in those patients. Furthermore, Brown *et al*. were able to reveal helicopter transport as an independent survival factor. In contrast, Talving *et al*. demonstrated an increased overall length of stay without prolonged intensive care treatment in HEMS patients [37]. As no survival benefit was measured in that study, the authors concluded that helicopter transport might only raise treatment duration without improving outcome. However, as the injury severity was significantly lower (HEMS 11.2 vs. GEMS 6.7) compared to the presented study (HEMS 26.0 vs. GEMS 23.5), as well as the NTBD evaluation, comparability of the results might be limited.

The present study also has its limitations. Although databank analyses represent a large number of patients, their validity is restricted due to detection of minor statistical differences without mandatory clinical relevance. Furthermore, we had to exclude approximately 6% due to missing data referring to the transportation mode. Although this might have influenced our results, we expect this bias to be of minor effect. In comparison, Galvagno excluded 40% due to missing disposition information. However, another bias could be expected by influencing factors not evaluated by the databank (weather conditions, transportation distances and so on). Further criticism could be offered due to the inclusion criteria of an ISS ≥9 points. We decided to use the inclusion criteria of ISS ≥9 because multiple patients with an ISS between 9 and 15 were transported by helicopter. We intended to include a vast number of patients without excluding a considerable number of traumatized patients *a priori*. This has been done by Braithwaite *et al*. before including patients with an ISS of 0 to 15 points [44]. We are aware that most papers used the inclusion criteria of ISS larger than 15 to describe multiple traumatized patients. This description is widely accepted and we do not intend to argue this aspect. We, therefore, strictly described our study population not as 'multiply traumatized' but as 'traumatized' to avoid confusion. Interestingly, mean and median ISS parameters were larger than 15 in the presented study, though. However, the inclusion criteria of ISS ≥9 has been used before in order to include traumatized patients [45-47].

Despite these limitations, the present study presents a large sample size evaluating preclinical as well as clinical parameters in order to reveal potential benefits of HEMS compared to GEMS rescue in traumatized patients.

## Conclusions

In conclusion, the present study demonstrates that HEMS rescue has its merit on traumatized patients. Despite an increased injury severity and a higher incidence of clinical complications, HEMS has a beneficial impact on survival. The survival benefit remained regardless of the subsequent treatment at level I trauma centers. HEMS physicians performed more invasive treatment on-scene but an expected increased accuracy of suspected diagnosis leading to correct triaging could not be proven. Further investigations emphasizing special subgroups and triage criteria might help to explain the demonstrated survival benefit.

## Key messages

• Transportation by HEMS resulted in a significant survival benefit compared to GEMS patients despite increased injury severity and incidence of posttraumatic complications (MODS, sepsis).

• The accuracy of prehospital documented diagnoses was not increased in HEMS compared to GEMS rescue.

• The extent of preclinical management was more extensive in HEMS resulting in prolonged on-scene times.

• HEMS patients were more often admitted to level I trauma centers.

## Abbreviations

ACCP: American College of Chest Physicians; AIS: Abbreviated Injury Scale; AUC: Akademie der Unfallchirurgie; CI: Confidence intervals; DGU^®^: German Society for Trauma Surgery; GCS: Glasgow Coma Scale; GEMS: Ground emergency medical services; GmbH: Limited Liability Companies Act; HEMS: Helicopter emergency medical services; ICU: Intensive Care Unit; IQR: Interquartile range; ISS: Injury Severity Score; MODS: Multiple Organ Dysfunction Syndrome; MTOS: Major Trauma Outcomes Study; OR: Odds ratio; RISC: Revised Injury Severity Classification; SCCM: Society of Critical Care Medicine; SD: Standard deviation; SMR: Standardized Mortality Ratio; SOFA: Sequential Organ Failure Assessment; SPSS: Statistical Package for the Social Sciences; TRISS: Trauma and Injury Severity Score; TR-DGU: TraumaRegister DGU^® ^of the German Society for Trauma Surgery.

## Competing interests

Each author certifies that he has no commercial association that might pose a conflict of interest with his scientific work. Research funding was provided by Deutsche Rettungsflugwacht, Filderstadt, Germany, to Christian Krettek, MD, solely. The authors declare that they have no competing interests.

## Authors' contributions

HA conceived this study, designed the trial, provided statistical advice on study design, analyzed the data and drafted the manuscript. RL provided statistical advice on the study design, analyzed the data and supervised the conduct of the trial and data collection. MF, PM, CZ and KR conceived the study and designed the trial. CK conceived the study, designed the trial, obtained research funding and supervised the conduct of the trial. FH conceived the study, designed the trial, obtained research funding, supervised the conduct of the trial and data collection, provided statistical advice on study design and analyzed the data. HA takes responsibility for the article as a whole. All authors contributed substantially to manuscript revision. All authors have read and approved the final manuscript for publication.
